# The Influence of Fiber on the Mechanical Properties of Geopolymer Concrete: A Review

**DOI:** 10.3390/polym15040827

**Published:** 2023-02-07

**Authors:** Tao Wang, Xiangqian Fan, Changsheng Gao, Chiyu Qu, Jueding Liu, Guanghui Yu

**Affiliations:** State Key Laboratory of Hydrology-Water Resources and Hydraulic Engineering, Nanjing Hydraulic Research Institute, Nanjing 210024, China

**Keywords:** fiber, geopolymer concrete, mechanical properties, fiber-reinforced, fracture toughness

## Abstract

Geopolymer is widely used as a supplement to cementitious composites because of its advantages of low carbon and environmental protection, and geopolymer concrete is also broadly used in practical engineering. In recent years, geopolymer concrete has attracted increasing interest owing to its superior mechanical properties, and a series of research results have been obtained. In this paper, from the preparation of geopolymer concrete, based on the characteristics that geopolymer concrete is brittle and easy to crack, the types and basic properties of fibers to enhance the toughness of concrete are analyzed, the advantages and disadvantages of different fibers used as a material to enhance the toughness of concrete are summarized, and we review the effects of type, shape, volume rate, aspect ratio, and hybrid fiber combinations on the static mechanical properties. The results indicate that fibers have significant potential to enhance the compressive strength, splitting tensile strength, flexural strength, and fracture toughness of geopolymer concrete, and the optimal fiber volume rate seems to be related to the fiber type. Whereas the effect of aspect ratio and hybrid fiber combinations on the properties of geopolymer concrete seems to be obvious. This paper reviews the influence of fiber on the basic mechanical properties of geopolymer concrete, which provides a solid foundation to promote the further development and application of the research on the toughness of fiber-reinforced geopolymer concrete and provides recommendations for future research.

## 1. Introduction

With good mechanical properties, traditional concrete materials are regarded as irreplaceable construction materials. Ordinary Portland cement serves as the main raw material for the preparation of concrete; however, a large number of greenhouse gases and harmful emissions are generated in the entire production process of cement generates [1], including carbon dioxide and sulfur dioxide [2]. It is reported that the production of one ton of cement will release one ton of carbon dioxide, and the carbon dioxide released by the production of cement accounts for about 5–7% of the total global carbon dioxide emissions [3,4,5,6]. Additionally, cement manufacturing requires continuous electrical energy, and its energy consumption is second only to steel and aluminum [7], which exerts enormous pressure on energy consumption [8]. Currently, countries around the world are increasingly concerned about the environmental sustainability of the construction industry. It is suggested that cement can be replaced with low-carbon green materials and reduce the cement proportion in concrete while maintaining the mechanical properties of concrete [9].

The use of geopolymer concrete can reduce carbon dioxide emissions and energy consumption in cement production and utilization in the construction industry [10,11]. With the deepening of the research on geopolymer, “geopolymer binder”, a new inorganic cementing material, has greatly developed and been applied in concrete in recent years [12]. The application can effectively reduce environmental pollution in the manufacturing process of “cementitious composites” [13] and maintain the original mechanical properties of concrete [14].

**Figure 1:**
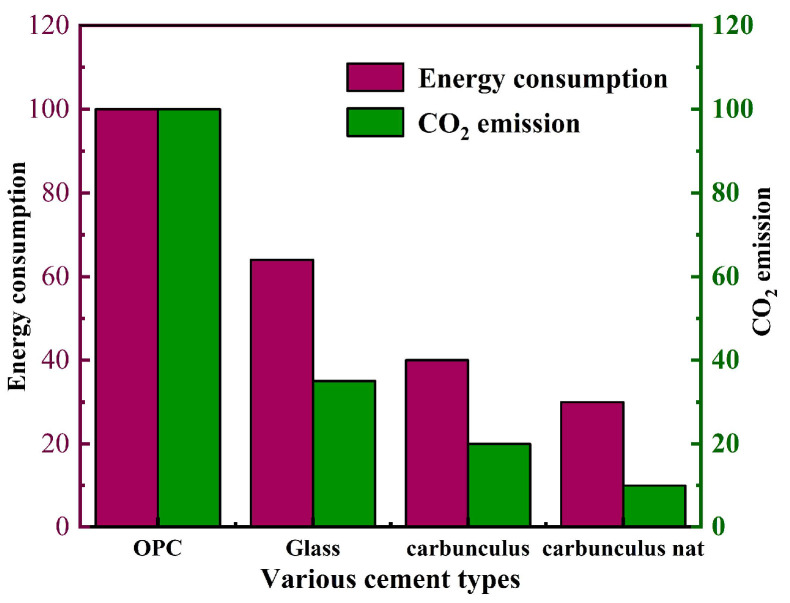
Comparison of energy consumption and CO_2_ emissions between ordinary Portland cement and geopolymer.

The research shows that the carbon dioxide emission of geopolymer concrete manufacturing is far lower than that of ordinary Portland cement concrete because the raw materials of geopolymer concrete do not need high-temperature calcination [15,16]. In addition, the energy consumption for the production of geopolymer concrete is greatly decreased. For example, compared with ordinary Portland cement concrete with the same strength, the energy consumption of fly ash-based geopolymer concrete is reduced by 70% [17]. The comparison of energy consumption and CO_2_ emissions of ordinary Portland cement and geopolymer is shown in Figure 1 [18]. Figure 1 shows that the CO_2_ emissions and energy consumption of geopolymers are much lower than ordinary Portland cement.

**Figure 2:**
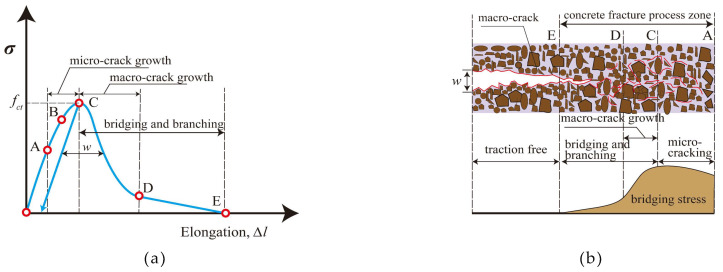
Schematic of the crack development process: (**a**) Stress-elongation curve of concrete; (**b**) fracture propagation diagram.

Compared with ordinary Portland cement concrete, geopolymer concrete not only has favorable environmental friendliness but also has high early strength, high durability, and low shrinkage [19,20,21]. However, as the research progressed, scholars have found that geopolymer concrete also has some disadvantages similar to ordinary concrete, such as high brittleness, poor toughness, and poor crack resistance [22,23]. Due to the long-term effect of the external environment, cracks often appear in geopolymer concrete [24]. Further expansion of cracks will reduce the bearing capacity and durability of structures or components, affect the appearance and service life of concrete structures, and even threaten people’s lives and property safety [25]. Therefore, the research on improving the toughness of geopolymer concrete is gradually obtaining broad attention. The formation process of cracks is shown in Figure 2 [26].

**Figure 3:**
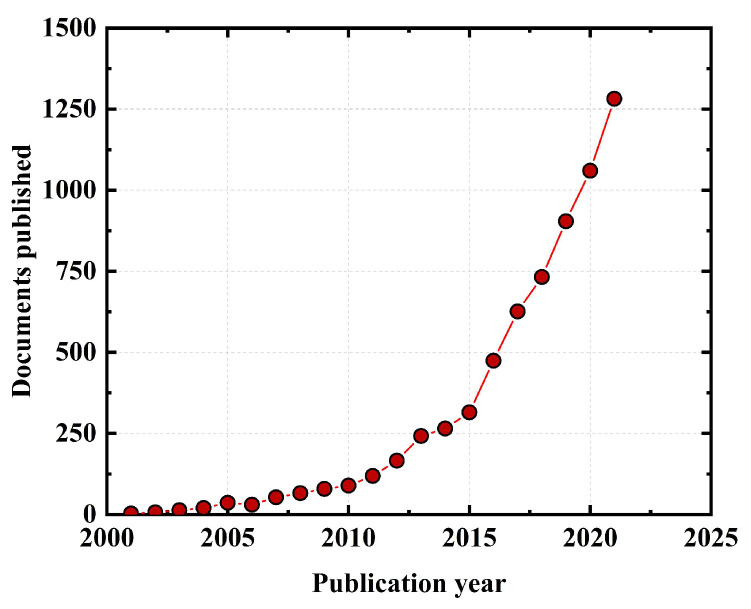
Quantity of papers on fiber-reinforced geopolymer concrete annually published in Science Direct.

In recent years, the effect of various types of fibers on the mechanical properties of geopolymer concrete has been the subject of many studies [27,28,29]. Figure 3 shows the number of papers on fiber-reinforced geopolymer concrete published annually in Science Direct [30]. As can be seen from Figure 3, there was less research on fiber-reinforced geopolymer concrete before 2010. Since 2018, the number of research papers has increased significantly, indicating that global scholars have paid more attention to this field. The research results show that the addition of fibers can effectively reduce the generation and expansion of cracks and improve the brittleness, crack resistance, flexural strength, and toughness of geopolymer concrete [31,32]. Accordingly, many scholars have carried out a variety of research on the basic mechanical properties and fracture properties of fiber-reinforced geopolymer concrete and have achieved some results.

Although several review articles have been published in the research area of geopolymer composites, they are focused on presenting research works regarding the development history, engineering applications, and durability of geopolymer concrete. The research on fiber-reinforced geopolymer concrete and improving its performance must be supplemented. Therefore, based on the research on the mechanical properties of geopolymer concrete in recent years, this paper reviews the preparation process of geopolymer concrete, analyzes the types and basic properties of fibers to improve the toughness of concrete, and puts forward the advantages and disadvantages of different fibers used as materials to enhance the toughness of concrete. By collecting experimental data from the published literature, a database of basic mechanical properties of fiber-reinforced geopolymer concrete with a relatively large sample size is established, and the effect of type, shape, volume rate, aspect ratio, and hybrid effect of fiber on the basic mechanical properties of geopolymer concrete is discussed. The purpose of this paper is to integrate the current research on fiber-reinforced geopolymer concrete and provide ideas for the green and sustainable development of geopolymer concrete in the future.

## 2. Preparation of Geopolymer Concrete

**Figure 4:**
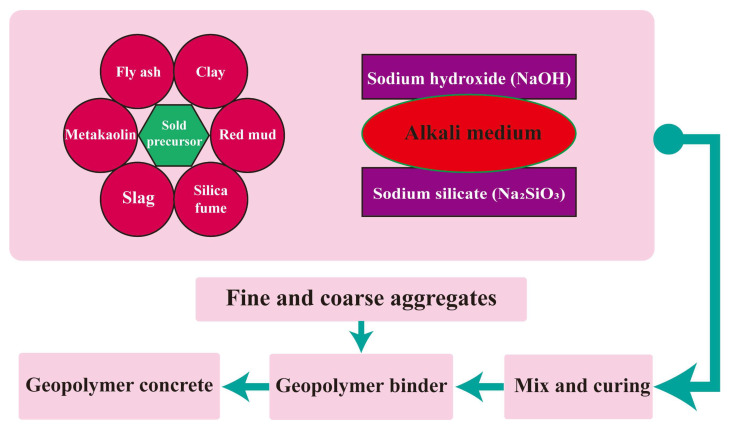
The preparation process of geopolymer concrete.

“Geopolymer”, first named by Davidovits in the late 1970s, is a green material synthesized from a variety of aluminosilicate materials rich in aluminum and silicon [11]. The aluminosilicate material can be obtained from fly ash, silica fume, metakaolin, palm oil fuel ash, and ground granulated blast furnace slag [33,34,35]. Geopolymer concrete is a new type of polymer concrete, which is polymerized by a geopolymer binder with an alkaline activator [36]. During the polymerization process, silicon and aluminum react rapidly under alkaline conditions, and then a three-dimensional polymerization chain of the Si–O–Al–O bond is generated [37]. The chemical reaction during the polymerization process is shown in Formula (1). The types of alkaline activators used in the polymerization process will significantly affect the mechanical properties of geopolymer concrete. Nowadays, the alkaline activators used mainly include the combination of sodium hydroxide (NaOH) and sodium silicate (Na_2_SiO_3_) and the combination of potassium hydroxide (KOH) and potassium silicate (K_2_SiO_3_) [38]. The preparation process of geopolymer concrete is shown in Figure 4 [39,40].


(1) $$\left\{ \begin{array}{l} {\left({\mathrm{Si}}_{2}O_{5}{\mathrm{Al}}_{2}O_{2}\right)}_{n}+H_{2}O+OH^{-}\to \mathrm{Si}{\left(\mathrm{OH}\right)}_{4}+\mathrm{Al}{\left(\mathrm{OH}\right)}^{4-}\\ \mathrm{Si}{\left(\mathrm{OH}\right)}_{4}+\mathrm{Al}{\left(\mathrm{OH}\right)}^{4-}\to {\left(\overset{|}{\underset{|}{\mathrm{Si}}}-O-\overset{|}{\underset{|}{\mathrm{Al}}}-O\right)}_{n}+4H_{2}O \end{array}\right.$$

The comparison of chemical composition between the geopolymer used currently and ordinary Portland cement is shown in Table 1.

**Figure 5:**
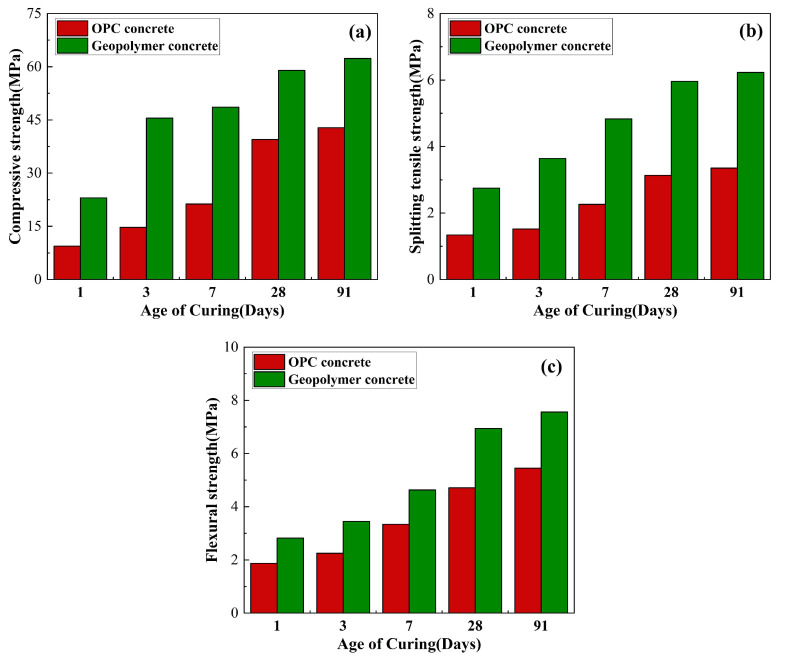
Comparison of mechanical properties between OPC concrete and geopolymer concrete: (**a**) Compressive strength; (**b**) splitting tensile strength; (**c**) flexural strength.

It can be seen from Table 1 that although the chemical composition of geopolymer is similar to that of ordinary Portland cement, the proportion of each chemical component is quite different. Additionally, different silica-aluminate material compositions have a great influence on the mechanical properties of geopolymer concrete [50]. The existing research results show that the performance of geopolymer concrete is superior to that of ordinary Portland cement concrete, and the performance difference is shown in Figure 5 [51].

## 3. Types of Fibers for Geopolymer Concrete Performance Enhancement

Generally speaking, the types of fibers used to improve the performance of geopolymer concrete can be divided into two categories, including artificial fiber and natural fiber [52]. Artificial fiber mainly includes steel fiber, inorganic fiber, and synthetic fiber; natural fiber mainly includes animal fiber, mineral fiber, and plant fiber. In order to solve the problem of brittleness and easy cracking of geopolymer concrete, adding different types of fibers into geopolymer concrete has become a common method in engineering applications.

### 3.1. Artificial Fiber

#### 3.1.1. Steel Fiber

**Figure 6:**
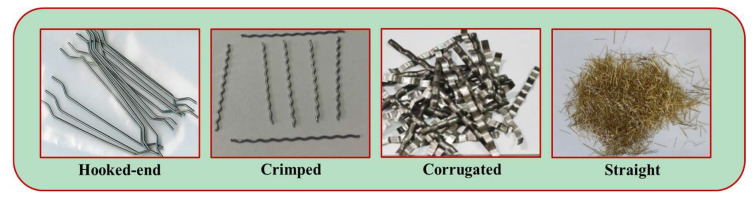
Typical types of steel fiber.

Steel fiber is mainly made of cold-drawn steel wire, steel plate, or other forms of steel, and its cross-sectional shape is mainly round, square, and rectangular [53,54]. Steel fiber is widely added to geopolymer concrete due to its high density, high tensile strength, and low cost [55]. In order to suppress the pulling of steel fibers from the concrete matrix, a large number of steel fibers with various shapes and geometries are manufactured worldwide [56]. The geometry of steel fibers that have been applied in geopolymer concrete includes hooked-end steel fiber, crimped steel fiber, straight steel fiber, twisted steel fiber, corrugated steel fiber, and others [57,58]; the more common types of steel fibers are shown in Figure 6.

The addition of steel fiber has been proven to help control the formation and development of cracks and can make geopolymer concrete, a brittle material, obtain higher crack resistance, and the benefits of using steel fiber to modify the properties of geopolymer concrete are mainly manifested in the following aspects [46,52]:

1. Improve the ductility, toughness, and flexural strength of cementitious materials;
2. Absorb energy, play the role of bridge connection between cracks, transfer loads;
3. Reduce shrinkage, creep, and permeability of concrete;
4. Improve the fatigue resistance and impact resistance of concrete.

In addition, the physical properties of steel fiber also affect the overall mechanical properties of steel fiber-reinforced geopolymer concrete. At present, the main characteristics of steel fibers used more in geopolymer concrete research are shown in Table 2. It can be seen from Table 2 that the length of steel fibers used more is generally between 10 mm and 35 mm, the diameter of steel fibers is between 0.12 mm and 0.56 mm, and the tensile strength of steel fibers is within the range of 900 MPa to 2670 MPa. The elastic modulus of steel fibers is mainly about 200 GPa, and the density of steel fibers used is generally about 7.85 g/cm^3^.

#### 3.1.2. Inorganic Fiber

**Figure 7:**
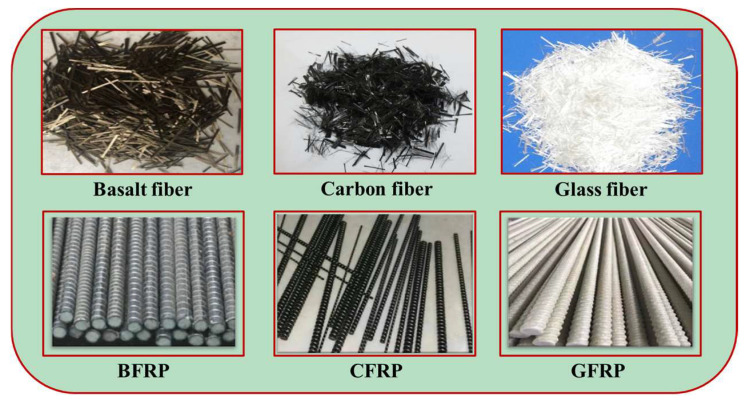
The types of inorganic fiber and fiber-reinforced polymer.

Inorganic fiber is a type of chemical fiber made from minerals. The main varieties are basalt fiber, glass fiber, and carbon fiber. Typical inorganic fibers and the types of polymer are shown in Figure 7.

Basalt fiber is composed of basalt gravel, which is environmentally friendly and has good mechanical properties [64]. Basalt fiber is produced in a similar way to glass fiber, but no chemicals are added in the production process, and the energy consumption is low. Based on the above characteristics, basalt fiber is widely used in various fields as an environmentally friendly material [65]. Additionally, basalt fiber has a three-dimensional molecular structure. Therefore, compared with one-dimensional linear polymer fibers, basalt fiber can improve the splitting tensile strength, flexural strength, and fracture toughness of concrete to a greater extent, and its improvement effect on the mechanical properties of concrete is better than those of polyethylene fiber and polypropylene fiber. Therefore, basalt fiber is widely used in concrete. The basic mechanical properties of typical basalt fibers are shown in Table 3.

Basalt fiber is also often used in the manufacture of basalt fiber-reinforced polymer (BFRP) bars [66]. With the advantages of a lightweight, high strength, and corrosion resistance, the BFRP bar serves as an ideal new material to replace steel to solve the problem of steel corrosion. Based on the above advantages, BFRP bars are often used as reinforcement bars in concrete structures to reduce the damage caused by steel corrosion. A typical picture of the BFRP bars used in the structures is shown in Figure 7.

Carbon fiber is a type of lightweight and high-strength inorganic fiber material, which is mainly composed of carbon atoms [67]. Over the years, carbon fiber has been widely used in concrete due to its advantages of high corrosion resistance, low density, high tensile strength, and high elastic modulus [30,68,69]. The basic mechanical properties of typical carbon fibers are shown in Table 3. It can be seen from Table 3 that the density of carbon fiber used more is between 1.70 g/cm^3^ and 1.82 g/cm^3^, the tensile strength of carbon fiber can reach 4558 MPa, and the elastic modulus of carbon fiber can reach about 230 GPa. Many studies have proved that the addition of carbon fiber into concrete can not only significantly improve its toughness, split tensile strength, and flexural strength [70] but also enhance its impact resistance and crack resistance [71,72].

Carbon fiber is also often used in the manufacture of carbon fiber-reinforced polymer (CFRP) [73]. A typical picture of CFRP bars used in engineering is shown in Figure 7 [74]. CFRP has the advantages of high stiffness, good durability performance, high tensile strength, and good corrosion resistance. In the field of building construction, CFRP is often used to modify and strengthen concrete structures [73,75].

Glass fiber is an inorganic non-metallic material with excellent performance. Its advantages are good insulation, strong heat resistance, good corrosion resistance, and high mechanical strength, but its disadvantages are brittleness and poor wear resistance [76]. The glass fiber is mainly made of pyrophyllite, quartz sand, limestone, dolomite, boracite, and boehmite through high-temperature melting, wire drawing, winding, weaving, and other processes. The basic mechanical properties of glass fiber commonly used in geopolymer concrete are shown in Table 3.

The research showed that glass fiber can eliminate microcracks in geopolymer concrete [77]. Therefore, glass fiber can be used to improve the toughness of geopolymer concrete to form a crack-free, high-toughness, and dense geopolymer matrix.

Glass fiber is also often used in the production of glass fiber-reinforced polymer (GFRP). The typical picture of GFRP used in the project is shown in Figure 7. The research showed that GFRP bars have good bonding ability with geopolymer concrete. Therefore, in the field of building construction, GFRP is often used to modify or strengthen concrete structures [78]. The capacity of geopolymer concrete specimens reinforced with GFRP can be increased by 68.5% at most.

**Table 3:** Basic mechanical properties of typical inorganic fibers.

Types	Length	Diameter	Tensile Strength	Elastic Modulus	Density	Ref.
	(mm)	(μm)	(MPa)	(GPa)	ρ/(g/cm^3^)	
Basalt	15	50	2830	83.9	2.70	[63]
	6	--	1450	88	2.63	[79]
Carbon	12	7	4000	242	--	[80]
	6	7	3950	238	1.70	[81]
	5–10	7	3530	230	--	[82]
	10	7	4558	231	1.82	[71]
Glass	6	20	1700	72	2.68	[81]
	12	12	2500	--	1.81	[61]
	6	100	1700	72	1.76	[83]

#### 3.1.3. Synthetic Fiber

**Figure 8:**
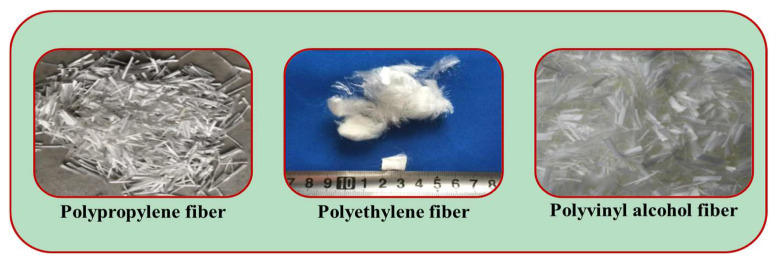
Typical types of synthetic fiber.

Synthetic fiber is mainly made of macromolecular compounds. In 1965, a researcher first proposed to apply synthetic fiber to cementitious composites [84]. In recent years, synthetic fiber has been widely concerned in the field of fiber-reinforced concrete with its low price, high corrosion resistance, lightweight, high strength, small diameter, and other advantages, and has been widely used in road, bridge, underground engineering, and other practical projects, which is an effective substitute for steel fiber and inorganic fiber. The addition of synthetic fiber into concrete can effectively prevent the development of concrete cracks and thus enhance the toughness of the material [85]. The types of synthetic fibers used to improve the toughness of concrete mainly include polyethylene (PE) fiber, polypropylene (PP) fiber, and polyvinyl alcohol (PVA) fiber [86]. The typical types of synthetic fibers are shown in Figure 8.

The properties of synthetic fibers have a great difference, especially in terms of tensile strength and modulus of elasticity, and the mechanical properties of several typical synthetic fibers are shown in Table 4. As can be seen from Table 4, the length of the synthetic fibers used more is generally between 3 mm and 50 mm, the diameter of the synthetic fibers used more is usually between 0.015 mm and 0.660 mm, the tensile strength of the synthetic fibers used more is within the range of 400 MPa and 3360 MPa. The elastic modulus of the synthetic fibers used more is mainly between 3.5 GPa and 115 GPa, and the density of the synthetic fibers used more is generally within the range of 0.91 g/cm^3^ and 1.30 g/cm^3^. Compared with steel fibers and inorganic fibers, the tensile strength and elastic modulus of synthetic fibers are lower. However, with the development of society, the processing technology of synthetic fibers is improving, the varieties of synthetic fibers are gradually increasing, the mechanical properties of synthetic fibers are also further optimized, and the development prospects of synthetic fibers will be better and better. The use of synthetic fibers to improve the properties of concrete can obtain higher economic benefits and better mechanical properties.

### 3.2. Natural Fiber

**Figure 9:**
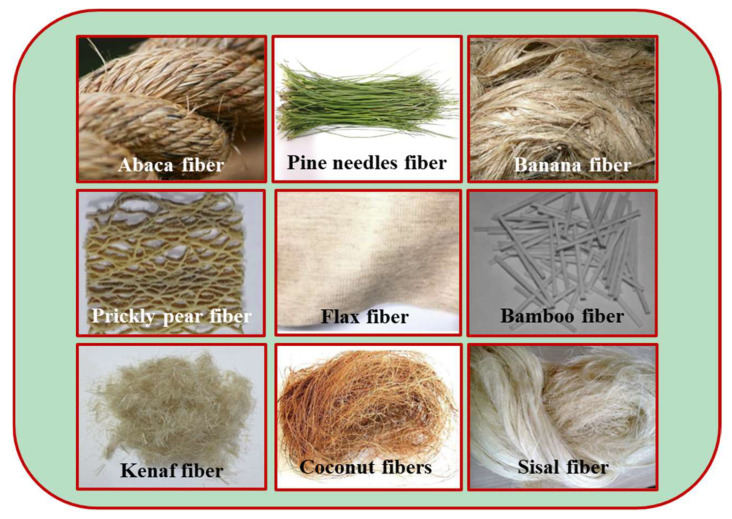
Typical types of plant fiber.

Natural fibers mainly come from three main sources: plant, animal, and mineral [90]. Plant fiber is a type of fiber obtained from different parts of the plant body, which is mainly composed of cellulose, hemicellulose, and lignin; animal fiber is a type of fiber obtained from animal hair, feathers, or insect secretions, which is mainly composed of proteins; mineral fiber is a type of fiber obtained from mineral rocks with fibrous structures, which is mainly composed of various oxides, such as silica and alumina. Compared to plant fibers, animal fibers are less used because animal fibers collected from animals are more difficult to be realized on a large scale. In addition, most mineral fibers have to be processed several times before application, and the only mineral fiber obtained without processing is asbestos, which is classified as a carcinogenic material and is not suitable for the preparation of environmentally friendly materials. In contrast, plant fiber, as an excellent natural polymer material, has the advantages of abundant reserves, repeated processing, and biodegradability [91]. Moreover, plant fiber has the mechanical characteristics of high strength and low hardness. Based on these advantages, adding plant fibers to concrete can improve brittleness and increase the toughness of the material [92]. Therefore, plant fibers have a wide application prospect in fiber-reinforced geopolymer concrete, and the typical plant fibers are shown in Figure 9.

**Figure 10:**
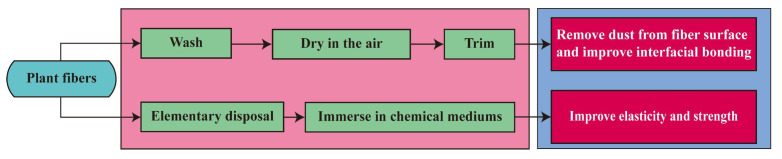
The general process of surface treatment of plant fibers.

Plant fiber is a biological resource, which is mainly composed of cellulose, pectin, and lignin [93]. Different compositions lead to different properties of fibers. When plant fibers are used as reinforcement material, the adhesion between the fibers and the matrix is affected by the hydrophobic or hydrophilic properties of the fibers, because plant fibers usually swell after contact with water. The property of plant fiber is an important factor in the performance of fiber-reinforced geopolymer concrete. To ensure the performance of fiber-reinforced geopolymer concrete, the surface of the plant fiber is usually pretreated. The general process of surface treatment of plant fibers is shown in Figure 10 [94].

The mechanical properties of plant fibers also depend on the physical characteristics of the fibers, such as diameter and length. The tensile strengths of plant fibers decrease with the increase in fiber length because longer fibers may have more defects and therefore may fail prematurely compared to shorter fibers. The basic mechanical properties of typical natural fibers are listed in Table 5 [95].

However, plant fibers also have the following limitations [96]:

1. The mechanical properties of plant fibers are lower compared with those of conventional fibers;
2. In an alkaline environment, plant fibers precipitate some carbohydrates, such as cellulose, hemicellulose, and lignin, which will inhibit the strength development of the gelling material;
3. The high water absorption and variability of plant fibers lead to poor adhesion of geopolymer-based gelling materials;
4. Plant fibers are easily degraded in alkaline matrices, which affects the long-term performance of the composites.

## 4. Mechanical Properties of Fiber-Reinforced Geopolymer Concrete

By combing the experimental data of the mechanical properties of fiber-reinforced geopolymer concrete in the published literature, the effects of fiber types on the compressive strength, splitting tensile strength, and flexural strength of geopolymer concrete are counted in Table 6. The fiber types counted in Table 6 mainly include steel fibers, glass fibers, basalt fibers, carbon fibers, polypropylene fibers, polyvinyl alcohol fibers, and plant fibers. The influence of the steel fiber shape on the compressive strength, splitting tensile strength, and flexural strength of geopolymer concrete is analyzed in Table 7. The steel fiber types in Table 7 are mainly straight, hooked, and crimped. The effects of fiber volume rate on compressive strength, splitting tensile strength, and flexural strength of geopolymer concrete are shown in Table 8. The fibers in Table 8 are mainly divided into synthetic fiber, inorganic fiber, steel fiber, and plant fiber. The influence of the fiber aspect ratio on the compressive strength, splitting tensile strength, and flexural strength of geopolymer concrete is counted in Table 9. The fiber aspect ratio in Table 9 is mainly 60, 70, 200, and 300. In Table 10, the effects of hybrid fibers on the compressive strength, splitting tensile strength, and h and flexural strength of geopolymer concrete are counted. The hybrid types are mainly replaced type and addition type.

### 4.1. Compressive Strength

#### 4.1.1. Fiber Type

**Figure 11:**
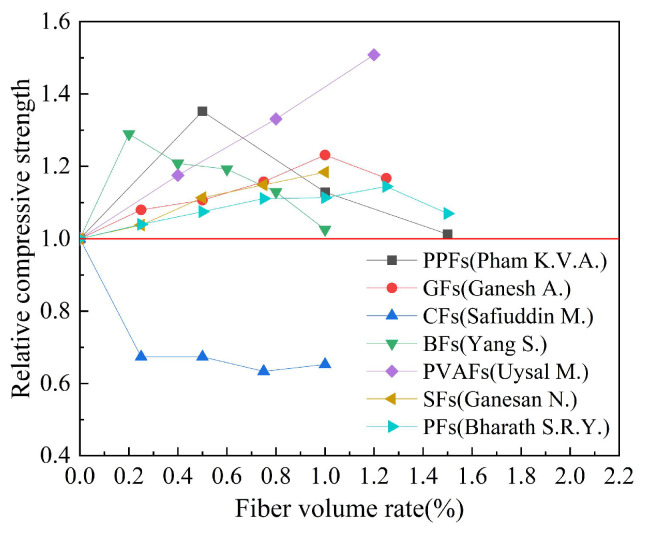
The effect of fiber type on compressive strength.

Based on the experimental data in Table 6, the effect of fiber type on the relative compressive strength of geopolymer concrete is studied. The results are shown in Figure 11. Relative compressive strength refers to the ratio of the compressive strength of fiber-reinforced geopolymer concrete to that of ordinary geopolymer concrete. It can be seen from Figure 11 that the optimal fiber volume rate of different types of fibers is not consistent, which is mainly due to the differences in physical properties, such as material density. We can observe that adding fibers can effectively improve the compressive strength of geopolymer concrete, among which PVAFs have the best effect on the compressive strength of geopolymer concrete. The results of Uysal M. et al. showed that when the volume ratio of PVAFs is 1.2%, the 28-day compressive strength of geopolymer concrete increased by 50%. It is worth noting that the results of Safiuddin M. et al. showed that the addition of CFs has a negative effect on the compressive strength of geopolymer concrete.

#### 4.1.2. Fiber Shape

**Figure 12:**
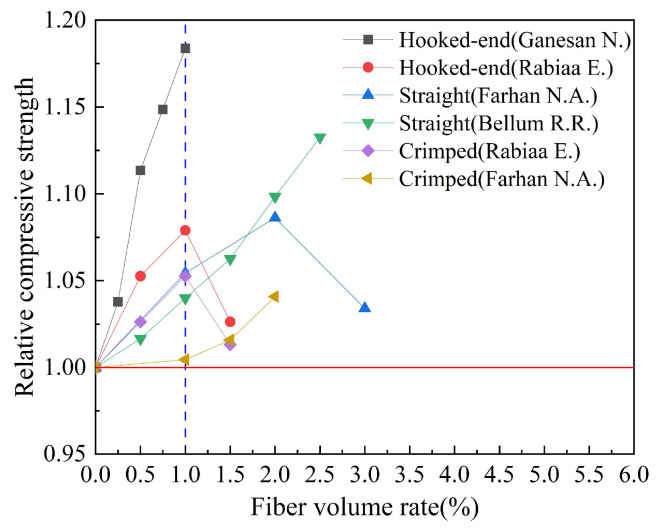
The effect of fiber shape on compressive strength.

Based on the experimental data in Table 7, the effect of steel fiber shape on the relative compressive strength of geopolymer concrete is analyzed. The results are shown in Figure 12. Relative compressive strength refers to the ratio of the compressive strength of fiber-reinforced geopolymer concrete to that of ordinary geopolymer concrete. We can observe that when the volume rate of steel fiber is between 0 and 1.0%, the compressive strength of geopolymer concrete increases with the increase in fiber volume rate. In addition, the hooked-end steel fiber has the best effect on the compressive strength of geopolymer concrete. The study of Ganesan N. et al. showed that when the volume rate of the hooked-end steel fiber is 1.0%, the relative compressive strength index of geopolymer concrete can reach 1.18.

#### 4.1.3. Fiber Volume Rate

**Figure 13:**
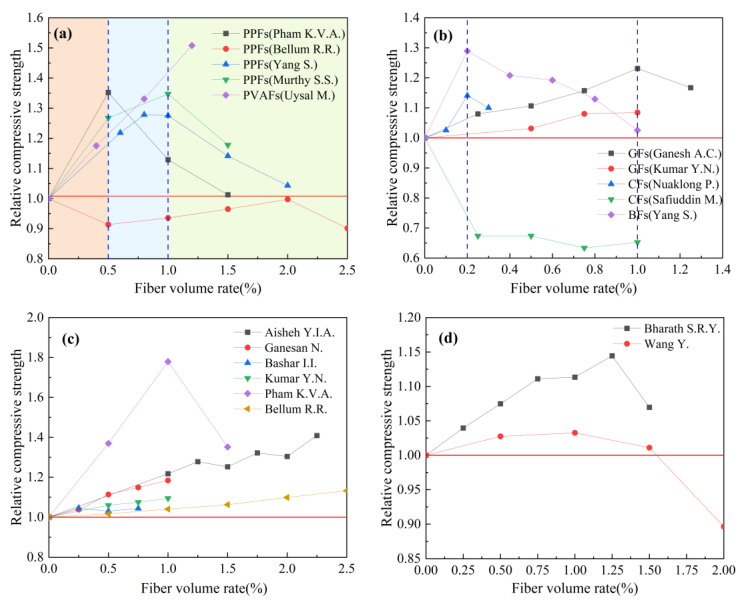
The effect of fiber volume rate on compressive strength: (**a**) Synthetic fiber; (**b**) inorganic fiber; (**c**) steel fiber; (**d**) plant fiber.

Based on the experimental data in Table 8, the effect of fiber volume rate on the relative compressive strength of geopolymer concrete is analyzed. The results are shown in Figure 13. Relative compressive strength refers to the ratio of the compressive strength of fiber-reinforced geopolymer concrete to that of ordinary geopolymer concrete.

Figure 13a summarizes the effect of synthetic fiber volume rate on the relative compressive strength of geopolymer concrete. The relevant research results showed that when the volume rate of synthetic fiber is between 0 and 0.5%, the compressive strength of geopolymer concrete will have a stable growth stage; when the volume rate of synthetic fiber is between 0.5% and 1.0%, the compressive strength of geopolymer concrete is relatively better, and its compressive strength is increased by 10% at least. In addition, the research results of Uysal M. et al. showed that when the volume rate of PVAFs is 1.25%, the compressive strength of PVAFs reinforced geopolymer concrete is 50.81% higher than that of ordinary geopolymer concrete. It is also worth noting that the results of Bellum R.R. et al. showed that the addition of PPFs has a negative effect on the compressive strength of geopolymer concrete.

Figure 13b summarizes the effect of the volume rate of inorganic fibers on the relative compressive strength of geopolymer concrete. The results of Ganesh A.C. et al. and Kumar Y.N. et al. showed that the enhancement effect on compressive strength of geopolymer concrete is the best when the volume rate of GFs is 1.0%. The experimental results of Yang S. et al. showed that the enhancement effect on the compressive strength of geopolymer concrete is the best when the volume rate of BFs is 0.2%. It is worth noting that Safiuddin M. et al. reported that the addition of CFs reduces the compressive strength of geopolymer concrete, which is contrary to the experimental results of Nuaklong P. et al.

Figure 13c summarizes the effect of steel fiber volume rate on the relative compressive strength of geopolymer concrete. It can be observed from Figure 13c that steel fiber can effectively improve the compressive strength of geopolymer concrete, and its reinforcement effect is better than other types of fibers. The experimental results of Pham K.V.A et al. showed that when the volume rate of steel fiber is 1.0%, the compressive strength of steel fiber reinforced geopolymer concrete increased by 77.91% compared with that of ordinary geopolymer concrete.

Figure 13d summarizes the effect of plant fiber volume rate on the relative compressive strength of geopolymer concrete. At present, there is relatively little relevant research. The research results of Bharath S.R.Y. et al. showed that the compressive strength of geopolymer concrete can be increased by 14.44% when the volume rate of plant fiber is 1.25%. However, the research results of Wang Y. et al. showed that the effect of plant fiber on the compressive strength of geopolymer concrete is not obvious. When the fiber volume rate is 1.0%, the compressive strength of geopolymer concrete is only increased by 2.75%. When the fiber volume rate is greater than 1.5%, the fiber is prone to agglomeration, which is not conducive to improving the compressive strength.

#### 4.1.4. Fiber Aspect Ratio

**Figure 14:**
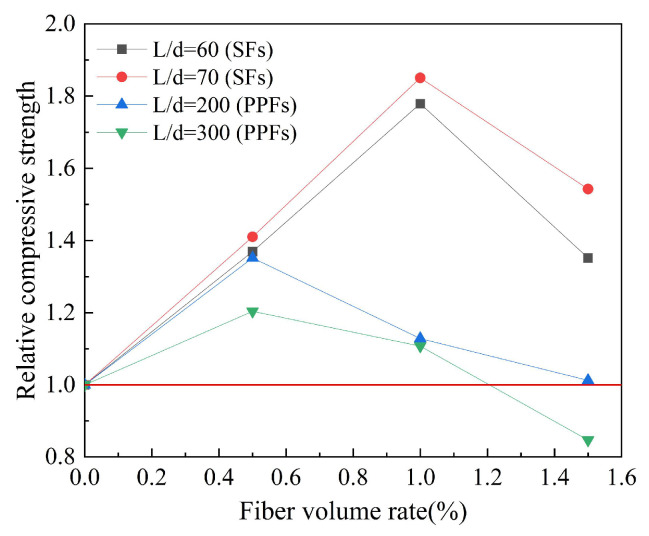
The effect of fiber aspect ratio on compressive strength.

Based on the experimental data in Table 9, the effect of the fiber aspect ratio on the compressive strength of geopolymer concrete is studied. The results are shown in Figure 14. Relative compressive strength refers to the ratio of the compressive strength of fiber-reinforced geopolymer concrete to that of ordinary geopolymer concrete. The fiber aspect ratio comprehensively considers the fiber length and diameter, but the optimal aspect ratio to improve the compressive strength needs further study. It can be seen from Figure 14 that for different types of fibers, the effect of the aspect ratio on the compressive strength of geopolymer concrete is not consistent. In the case of the optimal SFs volume rate, the increase in aspect ratio can improve the compressive strength of geopolymer concrete. In the case of the optimal PPFs volume rate, the increase in aspect ratio can reduce the compressive strength of geopolymer concrete.

#### 4.1.5. Fiber Hybrid Effect

**Figure 15:**
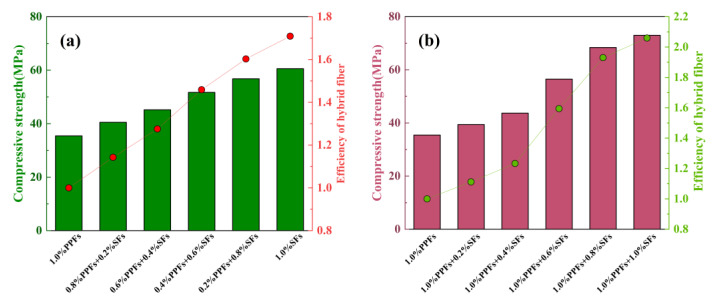
The effect of fiber hybrid effect on compressive strength: (**a**) The replacement type; (**b**) the addition type.

Based on the experimental data in Table 10, the effect of hybrid fibers on the compressive strength of geopolymer concrete is studied. The results are shown in Figure 15. The hybrid efficiency of fiber refers to the ratio of compressive strength of hybrid fiber-reinforced geopolymer concrete to that of single fiber-reinforced geopolymer concrete.

In the case of hybrid fiber-reinforced geopolymer concrete, for the replacement type, Figure 15a, when the base fiber (1.0% PPFs) is replaced by steel fiber with a volume rate increment of 0.2%, the results show that the compressive strength of geopolymer concrete increases with the increase in steel fiber replacement percentage. The compressive strength of hybrid fiber-reinforced geopolymer concrete begins to increase immediately after the addition of steel fiber. The hybrid fibers containing 0.2% PPFs and 0.8% SFs have the best reinforcement effect. The compressive strength of geopolymer concrete is 68.4 MPa, and the hybrid efficiency is 1.93. However, it should be noted that the compressive strength of all hybrid fiber-reinforced geopolymer concrete is lower than that of geopolymer concrete with a steel fiber volume rate of 1.0%. For the addition type, the results of adding steel fiber from 0.2% to 1.0% (in 0.2% increments) to base fiber (1.0% PPFs) reinforced geopolymer concrete are shown in Figure 15b. Similar to the replacement type, the compressive strength of the added hybrid fiber reinforced geopolymer concrete also shows an increasing trend with the increase in the percentage of steel fiber.

### 4.2. Splitting Tensile Strength

#### 4.2.1. Fiber Type

**Figure 16:**
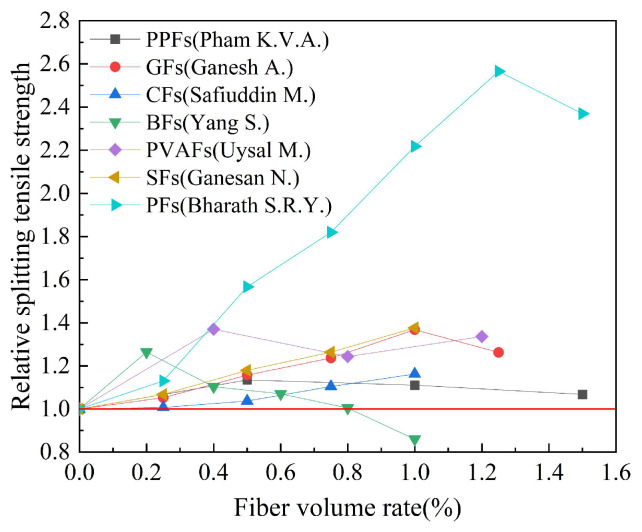
The effect of fiber type on splitting tensile strength.

Based on the experimental data in Table 6, the effect of fiber type on the relative splitting tensile strength of geopolymer concrete is studied. The results are shown in Figure 16. Relative splitting tensile strength refers to the ratio of the splitting tensile strength of fiber-reinforced geopolymer concrete to that of ordinary geopolymer concrete.

It can be seen from Figure 16 that similar to the influence of fibers on the relative compressive strength index of geopolymer concrete, various types of fibers have a better improvement effect on the splitting tensile strength of geopolymer concrete. Bharath S.R.Y. et al. found that plant fiber had the best splitting tensile strength improvement effect at a volume rate of 1.25%, and the relative splitting tensile strength index of geopolymer concrete was as high as 2.57. It is worth noting that Yang S. et al. found that BFs with a fiber volume rate greater than 0.8% had a negative effect on the splitting tensile strength of geopolymer concrete.

#### 4.2.2. Fiber Shape

**Figure 17:**
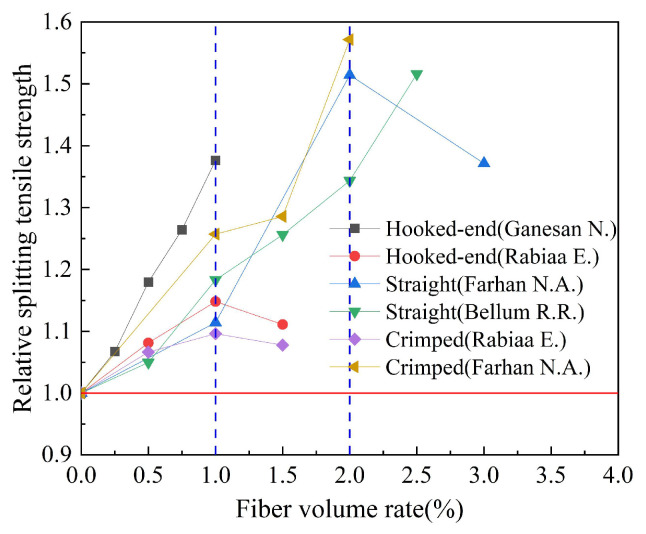
The effect of fiber shape on splitting tensile strength.

Based on the experimental data in Table 7, the influence of fiber shape on the relative splitting tensile strength of geopolymer concrete is studied. The results are shown in Figure 17. Relative splitting tensile strength refers to the ratio of the splitting tensile strength of fiber-reinforced geopolymer concrete to that of ordinary geopolymer concrete. We can observe that when the optimal fiber volume rate is 1.0%, the hooked-end steel fiber has a better effect on the splitting tensile strength of geopolymer concrete than the crimped steel fiber. At the same time, the research results of Farhan N.A. et al. showed that when the optimal fiber volume rate was 2.0%, the crimped steel fiber had a better effect on the splitting tensile strength of geopolymer concrete than the straight steel fiber.

#### 4.2.3. Fiber Volume Rate

**Figure 18:**
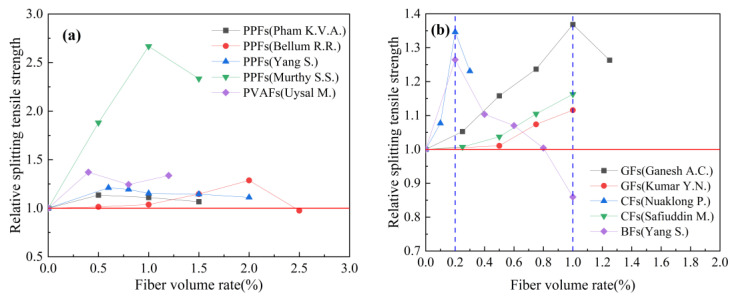

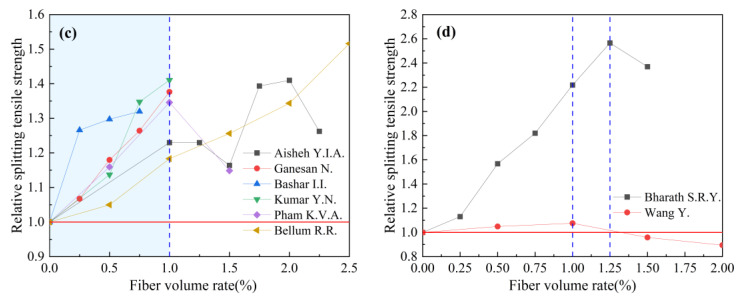
The effect of fiber volume rate on splitting tensile strength: (**a**) Synthetic fiber; (**b**) inorganic fiber; (**c**) steel fiber; (**d**) plant fiber.

Based on the experimental data in Table 8, the effect of the fiber volume rate on the relative splitting tensile strength of geopolymer concrete is studied. The results are shown in Figure 18. Relative splitting tensile strength refers to the ratio of the splitting tensile strength of fiber-reinforced geopolymer concrete to that of ordinary geopolymer concrete. Similar to the effect of the fiber volume rate on the relative compressive strength index of geopolymer concrete, the fiber volume rate has a better improvement effect on the relative splitting tensile strength index of geopolymer concrete.

Figure 18a summarizes the effect of the synthetic fiber volume rate on the relative splitting tensile strength index of geopolymer. From Figure 18a, it can be seen that the volume rate of synthetic fibers is generally between 0 and 2.0%. The research results of Murthy S.S. et al. showed that when the volume rate of PPFs is 1.0%, the relative splitting tensile strength index of geopolymer concrete can reach 2.67. Figure 18b summarizes the effect of the volume rate of inorganic fibers on the relative splitting tensile strength index of geopolymer concrete. It can be observed from Figure 18b that the volume rate of inorganic fibers is generally between 0 and 1.0%, which is generally smaller than that of synthetic fibers. Ganesh A.C. et al. obtained the best relative splitting tensile strength index of 1.36 when the volume rate of GFs is 1.0%. It is worth noting that the experimental results of Yang S. et al. showed that when the volume rate of BFs is greater than 0.8%, it has a negative effect on the splitting tensile strength of geopolymer concrete. Figure 18c summarizes the effect of SFs volume rate on the relative splitting tensile strength index of geopolymer concrete. It can be seen from Figure 18c that when the SFs volume rate is within the range of 0 to 1.0%, the splitting tensile strength of geopolymer concrete is gradually increased with the increase in the SFs volume rate. Figure 18d summarizes the effect of PFs volume rate on the relative splitting tensile strength index of geopolymer concrete. The research results of Bharaty S.R.Y. et al. showed that PFs can effectively improve the splitting tensile strength of geopolymer concrete. When the PFs volume rate is 1.25%, the relative splitting tensile strength index can reach 2.36. It is worth noting that the research results of Wang Y. et al. showed that the effect of PFs on the splitting tensile strength of geopolymer concrete is not obvious. The relative splitting tensile strength of different studies to enhance the best effect is different; this may be because the fiber enhancement effect is affected by other factors, such as material composition and curing time.

#### 4.2.4. Fiber Aspect Ratio

**Figure 19:**
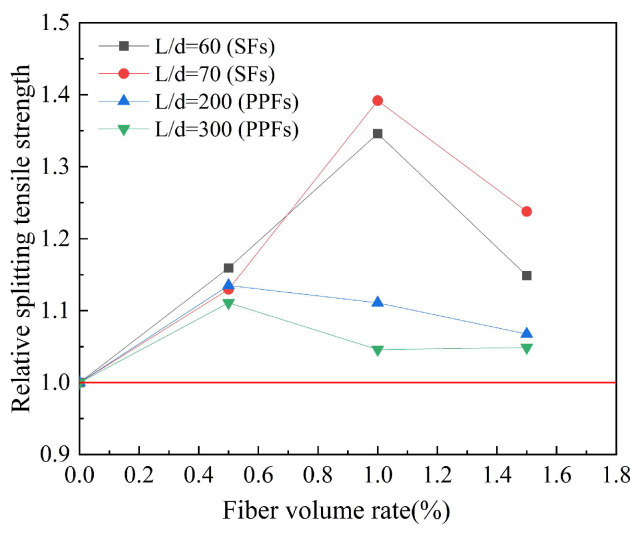
The effect of fiber aspect ratio on splitting tensile strength.

Based on the experimental data in Table 9, the effect of the fiber aspect ratio on the splitting tensile strength of geopolymer concrete is studied. The results are shown in Figure 19. Relative splitting tensile strength refers to the ratio of the splitting tensile strength of fiber-reinforced geopolymer concrete to that of ordinary geopolymer concrete. The effect of the fiber aspect ratio on the splitting tensile strength of geopolymer concrete is obvious. For SFs, in the case of the optimal fiber volume rate, the larger the fiber aspect ratio, the better the splitting tensile strength of steel fiber-reinforced geopolymer concrete. For PPFs, in the case of the optimal fiber volume rate, the smaller the fiber aspect ratio, the better the effect of PPFs on the splitting tensile strength of geopolymer concrete.

#### 4.2.5. Fiber Hybrid Effect

**Figure 20:**
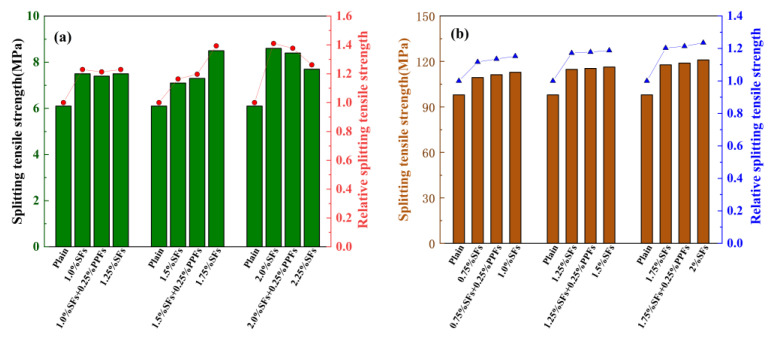
The effect of fiber hybrid effect on splitting tensile strength: (**a**) Data provided by [44]; (**b**) data provided by [43].

Based on the experimental data in Table 10, the effect of hybrid fibers on the splitting tensile strength of geopolymer concrete is studied. The results are shown in Figure 20. Relative splitting tensile strength refers to the ratio of the splitting tensile strength of fiber-reinforced geopolymer concrete to that of ordinary geopolymer concrete. As shown in Figure 20, Aisheh Y.I.A. et al. and Mousavinejad S.H.G. et al. studied the effect of the combination of SFs and PPFs on the splitting tensile strength of geopolymer concrete. The results showed that the combined use of SFs and PPFs can improve the splitting tensile strength of geopolymer concrete.

### 4.3. Flexural Strength

#### 4.3.1. Fiber Type

**Figure 21:**
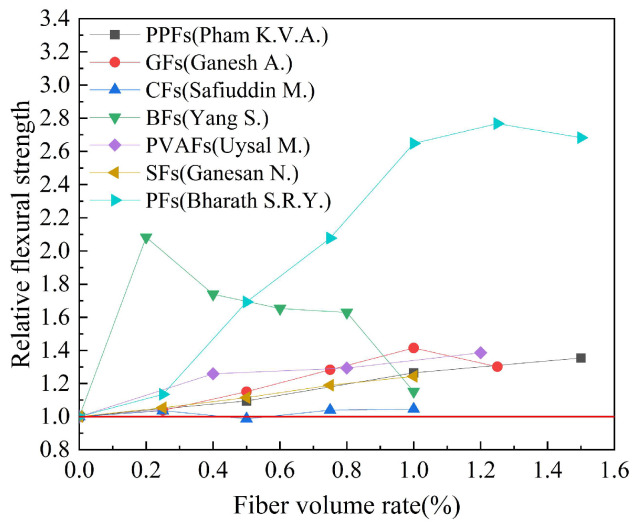
The effect of fiber type on flexural strength.

Based on the experimental data in Table 6, the effect of fiber type on the flexural strength of geopolymer concrete is studied. The results are shown in Figure 21. Relative flexural strength refers to the ratio of flexural strength of fiber-reinforced geopolymer concrete to that of ordinary geopolymer concrete. We can observe that various types of fibers have a certain effect on the flexural strength of geopolymer concrete, and BFs and PFs have a better effect on the flexural strength of geopolymer concrete. The experimental results of Yang S. et al. showed that BFs had the best flexural strength improvement effect at a volume rate of 0.2%, and the relative flexural strength index of geopolymer concrete was as high as 2.08. The experimental results of Bharath S.R.Y. et al. showed that PFs had the best flexural strength improvement effect at a volume rate of 1.25%, and the relative flexural strength index of geopolymer concrete was as high as 2.77. It is worth noting that the research results of Safiuddin M. et al. showed that the effect of CFs on the flexural strength of geopolymer concrete is not obvious. In the case of the optimal CFs volume rate, the relative flexural strength index of geopolymer concrete is only 1.04.

#### 4.3.2. Fiber Shape

**Figure 22:**
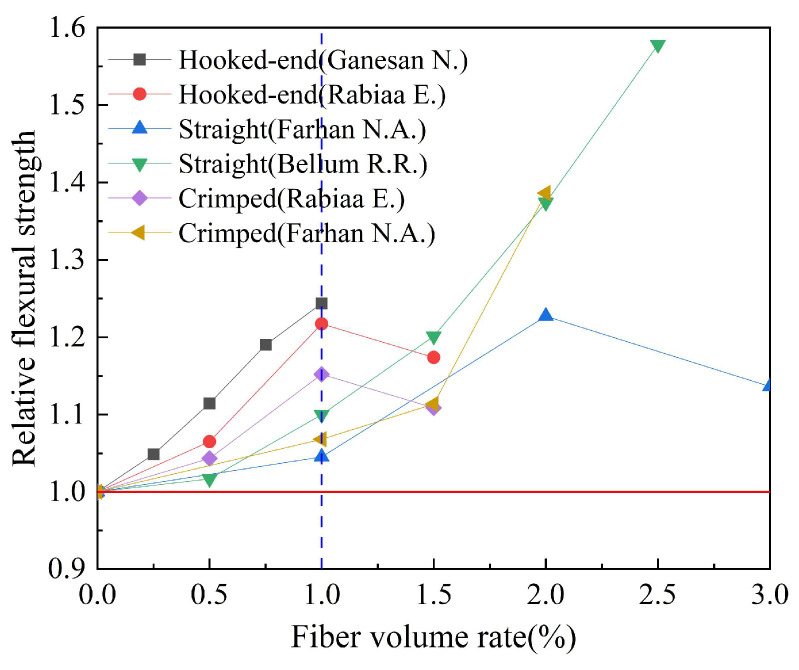
The effect of fiber shape on flexural strength.

Based on the experimental data in Table 7, the influence of the steel fiber shape on the flexural strength of geopolymer concrete is studied. The results are shown in Figure 22. Relative flexural strength refers to the ratio of flexural strength of fiber-reinforced geopolymer concrete to that of ordinary geopolymer concrete. We can observe that the relative flexural strength index of geopolymer concrete is increased with the increase in fiber volume rate within the range of 0 to 1.0%. The research results of Ganesan N. et al. and Rabiaa E. et al. showed that compared with other types of steel fibers, the hooked-end steel fiber had a better effect on the flexural strength of geopolymer concrete.

#### 4.3.3. Fiber Volume Rate

**Figure 23:**
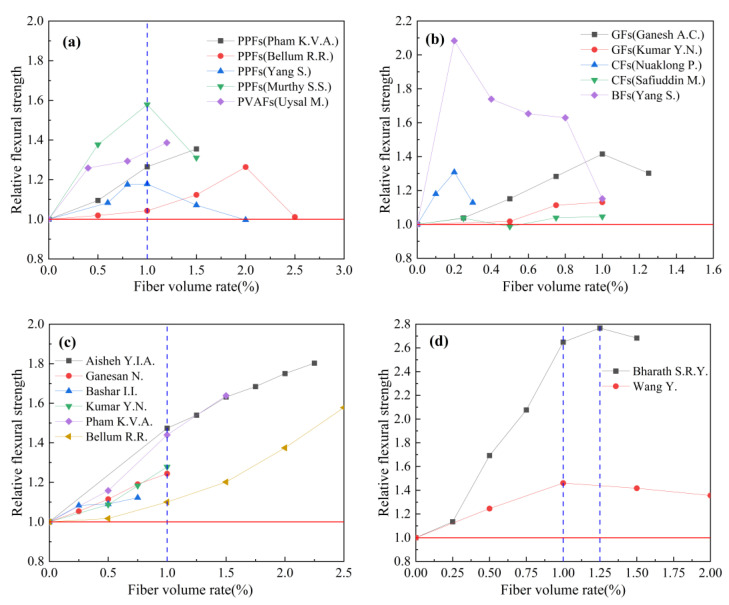
The effect of fiber volume rate on flexural strength: (**a**) Synthetic fiber; (**b**) inorganic fiber; (**c**) steel fiber; (**d**) plant fiber.

Based on the experimental data in Table 8, the effect of fiber volume rate on the relative flexural strength of geopolymer concrete is studied. The results are shown in Figure 23. Relative flexural strength refers to the ratio of flexural strength of fiber-reinforced geopolymer concrete to that of ordinary geopolymer concrete.

Figure 23a summarizes the effect of the synthetic fiber volume rate on the relative flexural strength index of geopolymer concrete. The relevant research results showed that the flexural strength of geopolymer concrete is gradually improved with the increase in fiber volume rate in the range of 0 to 1.0%. When the volume rate of synthetic fiber exceeds 1.0%, its effect on the flexural strength of geopolymer concrete is weakened. Figure 23b summarizes the effect of the volume rate of inorganic fibers on the relative flexural strength index of geopolymer concrete. It can be observed that the volume rate of inorganic fibers is generally in the range of 0 to 1.0%. The results of Ganesh A.C. et al. and Kumar Y.N. et al. showed that a 1.0% volume rate of GFs had the best effect on improving the flexural strength of geopolymer concrete. The results of Nuaklong P. et al. showed that the volume rate change in CFs had an effect on the flexural strength of geopolymer concrete, while the results of Safiuddin M. et al. showed that the volume rate change in CFs had no obvious effect on geopolymer concrete. Figure 23c summarizes the effect of SFs volume rate on the relative flexural strength index of geopolymer concrete. We can observe that with the increase in SFs volume rate, the flexural strength of geopolymer concrete is gradually increased. Figure 23d summarizes the effect of PFs volume rate on the relative flexural strength index of geopolymer concrete. We can observe that the flexural strength of geopolymer concrete increases with the increase in fiber volume rate in the range of 0 to 1.0%.

#### 4.3.4. Fiber Aspect Ratio

**Figure 24:**
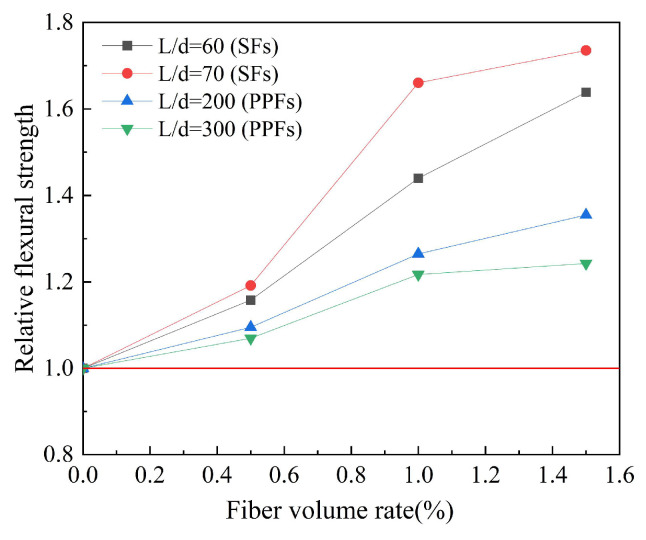
The effect of fiber aspect ratio on flexural strength.

Based on the experimental data in Table 9, the effect of the fiber aspect ratio on the relative flexural strength of geopolymer concrete is studied. The results are shown in Figure 24. Relative flexural strength refers to the ratio of the flexural strength of fiber-reinforced geopolymer concrete to that of ordinary geopolymer concrete. Similar to the results of splitting tensile strength, the effect of fiber aspect ratio on the flexural strength of geopolymer concrete is obvious. For SFs, in the case of the optimal fiber volume rate, the greater the fiber aspect ratio, the better the effect of SFs on the flexural strength of geopolymer concrete; for PPFs, in the case of the optimal fiber volume rate, the smaller the fiber aspect ratio, the better the effect of PPFs on the flexural strength of geopolymer concrete.

#### 4.3.5. Fiber Hybrid Effect

**Figure 25:**
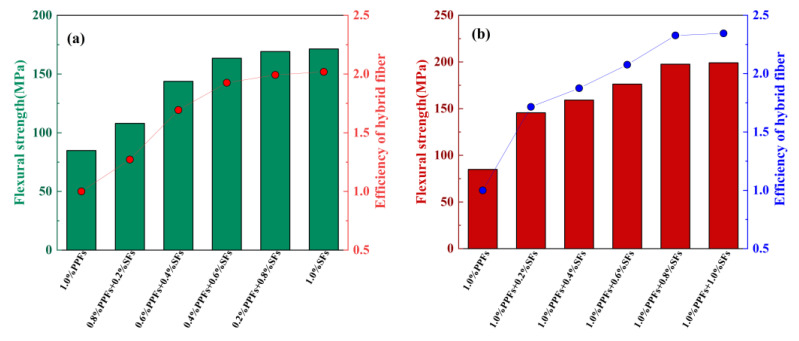
The effect of fiber hybrid effect on flexural strength: (**a**) The replacement type; (**b**) the addition type.

Based on the experimental data in Table 10, the effect of hybrid fibers on the relative flexural strength of geopolymer concrete is studied. The results are shown in Figure 25. Relative flexural strength refers to the ratio of the flexural strength of fiber-reinforced geopolymer concrete to that of ordinary geopolymer concrete. Similar to the compressive strength of hybrid fiber-reinforced geopolymer concrete, the flexural strength of hybrid fiber-reinforced geopolymer concrete also shows an increasing trend with the increase in steel fiber content.

## 5. Fracture Toughness of Fiber-Reinforced Geopolymer Concrete

**Figure 26:**
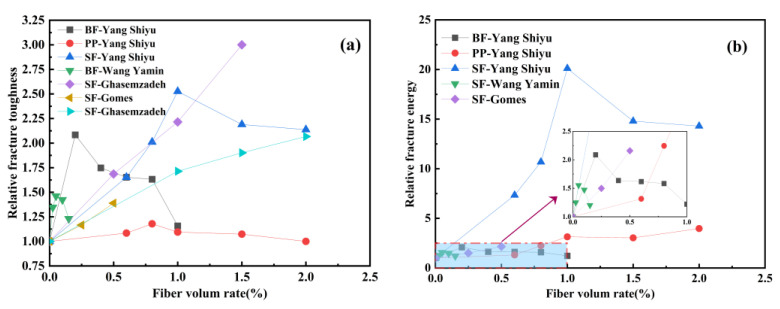
The effect of fiber volume rate on fracture properties of common geopolymer concrete: (**a**) Relative fracture toughness; (**b**) relative fracture energy.

From the perspective of engineering safety, the ideal damage mode of the structure should be accompanied by toughness damage with large deformation and high energy consumption. Geopolymer concrete has the disadvantage of being brittle and prone to cracking in use. Fibers, as a reinforcing material, have a more significant role in improving the brittleness and increasing the toughness of geopolymer concrete. In order to further understand the effect of fiber volume rate on the fracture toughness of geopolymer concrete, this paper analyzes the relative fracture toughness and relative fracture energy to eliminate the influence of different calculation methods. Based on the existing experimental research, the trend of the relative fracture toughness and relative fracture energy of geopolymer concrete with the fiber volume rate is shown in Figure 26.

According to the analysis of Figure 26a, Shiyu Yang et al. [43] found that the fracture toughness of geopolymer concrete was enhanced by basalt fiber, polypropylene fiber, and steel fiber, respectively, and the fracture toughness of geopolymer concrete showed a trend of first increasing and then decreasing with the increase in fiber volume rate, and the specimens containing 0.2% basalt fiber, 0.8% polypropylene fiber, and 1.0% steel fiber have the best reinforcement effect, among which basalt fiber has the best toughening effect. Meanwhile, the results of Wang Yamin et al. [79] also showed that the fracture toughness increased with the increase in basalt fiber volume rate in a certain range but decreased significantly when the fiber volume rate exceeded a certain value. Ghasemzadeh et al. [108,109] and Gomes et al. [23] showed that the addition of steel fiber could effectively enhance the fracture toughness of geopolymer concrete. The fracture toughness of geopolymer concrete increased with the increase in steel fiber volume rate.

According to Figure 26b, Shiyu Yang et al. [43] found that the fracture energy of basalt fiber and steel fiber-reinforced geopolymer concrete can be greatly improved with the increase in fiber volume rate within a certain range, and once the fiber volume rate exceeds the range, the fracture energy of the specimen begins to decrease. Similarly, the research results of Wang Yamin et al. [79] also showed that the fracture energy of geopolymer concrete first increased and then decreased with the increase in the basalt fiber volume ratio. However, when Shiyu Yang et al. [43] used polypropylene fibers as reinforcement material for geopolymer concrete, the fracture energy of the specimens gradually increased with the increase in fiber volume ratio; meanwhile, Gomes et al. [23] also obtained a consistent conclusion by analyzing the fracture energy of steel fiber-reinforced geopolymer concrete.

**Figure 27:**
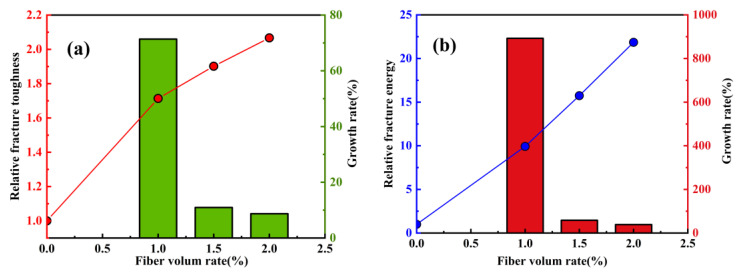
The effect of fiber volume rate on fracture properties of ultra-high-performance geopolymer concrete: (**a**) Relative fracture toughness; (**b**) relative fracture energy.

In addition, Ghasemzadeh et al. [109] studied the effect of fiber volume rate on the fracture properties of ultra-high performance geopolymer concrete (UHPGC), and the research results are shown in Figure 27. The results have shown that the addition of fibers can effectively enhance the fracture toughness and fracture energy of ultra-high-performance geopolymer concrete, and the improvement effect is obvious.

The above research showed that the addition of fibers can effectively enhance the fracture performance of geopolymer concrete. The main reason is that the bridging, pull-out, and fracture of fibers consumed more energy during the fracture process of fiber-reinforced geopolymer concrete. However, when the fiber volume rate exceeds a certain range, the formation of fiber clusters leads to the formation of internal defects in the geopolymer concrete, reducing the energy consumption to the extent that the fracture performance of some specimens decreases.

## 6. Conclusions

As a new type of composite material with the advantages of high strength, low carbon, and environmental protection, geopolymer concrete has been widely used in the repair and reinforcement of concrete structures in recent years. In order to overcome the disadvantages of geopolymer concrete, such as brittleness and susceptibility to cracking, research on fiber-reinforced geopolymer concrete has received extensive attention in recent years. Considering the space in the existing literature, this paper presents an extensive review of the basic mechanical properties and fracture toughness of fiber-reinforced geopolymer concrete, using a systematic review approach. The following conclusions are obtained.

1. Nowadays, the most used sources of silicate materials are mainly fly ash, silica fume, metakaolin, ground blast furnace slag, and slag, among which fly ash is mainly used. Under the same circumstances, geopolymer concrete performs better than ordinary Portland cement concrete in compressive strength, tensile strength, durability, fire resistance, CO_2_ emission, and setting time.
2. Many studies have shown that the addition of fiber-reinforced materials has a positive effect on the compressive strength of geopolymer concrete, but some researchers have come to the opposite conclusion. The main reason for this phenomenon is that the influence of fiber on the compressive strength of geopolymer concrete is less than that of the water-binder ratio, age, curing environment, and aggregate type.
3. The splitting tensile strength and flexural strength of geopolymer concrete are closely related to the type, shape, volume rate, and aspect ratio of fiber. PFs has a good effect on the splitting tensile strength and flexural strength of geopolymer concrete. The performance of hooked-end SFs is better under the same volume rate. In general, the fiber volume rate and aspect ratio that have the best effect on improving the splitting tensile strength and flexural strength of geopolymer concrete also depends on the type of fiber.
4. The combination of SFs and PPFs can improve the mechanical properties of geopolymer concrete. The compressive strength, splitting tensile strength, and flexural strength of geopolymer concrete show an increasing trend with the increase in steel fiber content.
5. The effect of fibers on the fracture toughness of geopolymer concrete is obvious. Within a certain range, the fracture toughness of geopolymer concrete increases with the increase in fiber volume rate; when the fiber volume rate exceeds a certain range, the fracture toughness of geopolymer concrete decreases with the increase in fiber volume rate.

## 7. Recommendations for Future Research

1. In this paper, based on the experimental data in the published literature, a basic mechanical properties test database of fiber-reinforced geopolymer concrete with a relatively large sample size is established. The database can be used to study the basic mechanical properties of geopolymers, and the results obtained can be used as a basis for further research on structural properties, numerical simulation, and fracture mechanics of geopolymer concrete.
2. This paper only analyzes the macroscopic mechanical properties of fiber-reinforced geopolymer concrete. A more comprehensive study on the microstructure of fiber-reinforced geopolymer concrete can be further conducted, and the relationship between macroscopic properties and the microstructure of fiber-reinforced geopolymer concrete can be established.
3. Fiber-reinforced geopolymer concrete has evolved from a new material to a successful and widely applied material due to its mechanical properties and advantages over conventional concrete, but the higher production cost of fiber-reinforced geopolymer concrete has limited its usage in the public works sector. Compared to normal concrete, the addition of fibers raises the production cost of fiber-reinforced geopolymer concrete. Thus, finding sustainable, environmentally friendly, and cost-reducing methods is one of the directions for further applications of fiber-reinforced geopolymer concrete.

## Figures and Tables

**Table 1:** Chemical composition of ordinary Portland cement and geopolymer (%).

Types	SiO_2_	Al_2_O_3_	Fe_2_O_3_	CaO	Na_2_O	K_2_O	SO_3_	TiO_2_	LOI	Ref.
OPC	19.69	5.16	2.88	62.12	0.17	0.88	2.63	--	2.99	[20]
FA	53.71	27.20	11.70	1.90	0.36	0.54	0.30	1.62	0.68	[41]
	51.11	25.56	12.48	4.30	0.77	0.70	0.24	1.32	0.57	[42]
	56.90	26.54	5.88	5.58	0.69	--	0.57	1.55	1.20	[43]
GGBFS	29.96	12.25	0.52	45.45	0.31	0.38	3.62	0.46	2.39	[41]
	27.42	14.00	1.00	44.27	0.52	0.89	1.72	2.48	2.30	[43]
	36.00	11.00	1.50	39.00	0.72	0.76	--	--	2.44	[44]
SF	94.40	1.32	0.87	0.49	0.31	1.01	--	--	2.50	[45]
	35.31	16.56	0.57	38.51	0.32	0.67	2.57	--	1.70	[46]
	95.10	1.22	0.95	0.59	0.42	1.20	--	--	2.61	[44]
MK	54.00	31.70	4.89	--	2.32	4.05	--	1.41	1.41	[47]
	35.31	16.56	0.57	38.51	0.32	0.67	2.57	--	1.77	[48]
	52.68	42.42	2.02	0.04	0.07	0.34	0.05	--	1.40	[49]
POFA	67.72	3.71	4.71	5.57	0.16	7.67	1.07	--	6.20	[49]

Key: OPC is ordinary Portland cement; FA is fly ash; SF is silica fume; MK is metakaolin; POFA is palm oil fuel ash; GGBFS is ground granulated blast furnace slag; LOI is loss on ignition.

**Table 2:** Basic mechanical properties of steel fibers.

Types	Length	Diameter	Tensile Strength	Elastic Modulus	Density	Ref.
	(mm)	(mm)	(MPa)	(GPa)	ρ/(g/cm^3^)	
Hooked end	13	0.20	2000	200	7.85	[3]
	30	0.56	1100	200	7.85	[59]
	35	0.55	1350	210	--	[60]
Crimped	25	0.50	900–1250	200–210	--	[52]
	25	0.50	2670	--	--	[61]
Straight	13	0.16	2500	200	7.90	[62]
	15	0.12	1200	200	7.80	[63]
	10	0.12	1200	200	7.80	[63]

**Table 4:** Basic mechanical properties of typical synthetic fibers.

Types	Length	Diameter	Tensile Strength	Elastic Modulus	Density	Ref.
	(mm)	(mm)	(MPa)	(GPa)	ρ/(g/cm^3^)	
PPFs	3–19	0.017	461	4.9	0.91	[3]
	6	0.035	400	3.5	0.91	[45]
	12	0.040	480	5.0	0.91	[87]
	6–50	--	310–600	3.5–6.5	0.91–0.95	[47]
PVAFs	12	0.020	1400–1600	35.0–39.0	1.26–1.29	[87]
	30	0.660	800	29.0	1.30	[59]
	12	0.015	1560	29.5	1.30	[88]
	12	0.040	1600	40.0	1.30	[89]
PEFs	--	0.020	2900	116	0.97	[64]
	6/12	0.022	3360	115	0.97	[6]

**Table 5:** Basic mechanical properties of natural fibers [95].

Types	Density	Tensile Strength	Elastic Modulus	Elongation
	(g/cm^3^)	(MPa)	(GPa)	%
Abaca fiber	1.50	400	12.00	3.0–10.0
Bamboo fiber	1.10	500	35.91	1.4
Banana leaf fiber	1.35	600	17.85	3.4
Coconut leaf fiber	1.15	500	2.50	20.0
Coconut shell fiber	1.20	175	4.00–6.00	30.0
Cotton fiber	1.60	287–597	5.50–12.60	7.0–8.0
Flax fiber	1.50	800–1500	27.60–80.00	1.2–3.2
Hemp fiber	1.48	550–900	70.00	2.0–4.0
Jute fiber	1.46	393–800	10.00–30.00	1.5–1.8
Red hemp fiber	1.45	930	53.00	1.6
Ramie fiber	1.50	220–938	44.00–128.00	2.0–3.8
Sisal fiber	1.45	530–640	9.4.00–22.00	3.0–7.0
Cork fiber	1.50	1000	40.00	4.4
Silk fiber	1.30	100–1500	5.00–25.00	15.0–60.0
Feather fiber	0.90	100–203	3.00–10.00	6.9
Wool fiber	1.30	50–315	2.30–5.00	13.2–35.0

**Table 6:** The effect of fiber type on geopolymer concrete.

Authors	Fiber Types	Fiber Volume Rate (%)	Compressive Strength (MPa)	Splitting Tensile Strength (MPa)	Flexural Strength (MPa)	Ref.
Pham K.V.A., et al.	PPFs	0–1.50	32.05–43.33	3.70–4.20	5.89–7.98	[97]
Ganesh A., et al.	GFs	0–1.25	41.3–50.85	3.80–5.20	5.30–7.50	[83]
Safiuddin M., et al.	CFs	0–1.00	60.20–95.00	4.30–5.00	7.60–8.05	[98]
Yang S., et al.	BFs	0–1.00	54.36–70.09	2.08–3.06	3.37–7.02	[43]
Uysal M., et al.	PVAFs	0–1.20	28.42–42.86	2.05–2.81	9.58–13.28	[99]
Ganesan N., et al.	SFs	0–1.00	37.00–43.80	3.56–4.90	4.10–5.10	[100]
Bharath S.R.Y., et al.	PFs	0–1.50	43.20–49.44	4.60–11.80	5.20–14.39	[39]

Key: PPFs is the polypropylene fibers; GFs is the glass fibers; CFs is the carbon fibers; BFs is the basalt fibers; PVAFs is the polyvinyl alcohol fibers; SFs is the steel fibers; PFs is the plant fibers.

**Table 7:** The effect of steel fiber shape on geopolymer concrete.

Authors	Fiber Shapes	Fiber Volume Rate (%)	Compressive Strength (MPa)	Splitting Tensile Strength (MPa)	Flexural Strength (MPa)	Ref.
Ganesan N., et al.	Hooked end	0–1.00	37.00–43.80	3.56–4.90	4.10–5.10	[100]
Rabiaa E., et al.	Hooked end	0–1.50	38.00–41.00	2.70–3.10	4.60–5.60	[101]
Farhan N.A., et al.	Straight	0–3.00	44.10–47.90	3.50–5.30	4.40–5.40	[102]
Bellum R.R., et al.	Straight	0–2.50	54.30–61.50	5.62–8.52	7.70–12.15	[103]
Rabiaa E., et al.	Crimped	0–1.50	38.00–40.00	2.70–2.96	4.60–5.30	[101]
Farhan N.A., et al.	Crimped	0–2.00	44.10–45.90	3.50–5.50	4.40–6.10	[102]

**Table 8:** The effect of fiber volume rate on geopolymer concrete.

Authors	Fiber Types	Fiber Volume Rate (%)	Compressive Strength (MPa)	Splitting Tensile Strength (MPa)	Flexural Strength (MPa)	Ref.
Pham K.V.A., et al.	PPFs	0–1.50	32.05–43.33	3.70–4.20	5.89–7.98	[97]
Bellum R.R., et al.	PPFs	0–2.50	48.93–54.30	5.48–7.23	7.70–9.73	[103]
Yang S., et al.	PPFs	0–2.00	54.36–69.49	2.42–2.93	3.36–3.97	[43]
Murthy S.S., et al.	PPFs	0–1.50	31.77–42.80	1.26–3.36	4.96–7.83	[104]
Uysal M., et al.	PVAFs	0–1.20	28.42–42.86	2.05–2.81	9.58–13.28	[99]
Ganesh A.C., et al.	GFs	0–1.25	41.30–50.85	3.80–5.20	5.30–7.50	[83]
Kumar Y.N., et al.	GFs	0–1.00	43.00–46.65	4.75–5.30	5.75–6.50	[77]
Nuaklong P., et al.	CFs	0–0.30	46.90–53.50	2.60–3.50	3.90–5.10	[105]
Safiuddin M., et al.	CFs	0–1.00	60.20–95.00	4.30–5.00	7.60–8.05	[98]
Yang S., et al.	BFs	0–1.00	54.36–70.09	2.08–3.06	3.37–7.02	[43]
Aisheh Y.I.A., et al.	SFs	0–0.25	115.00–162.00	6.10–8.60	7.60–13.70	[44]
Ganesan N., et al.	SFs	0–1.00	37.00–43.80	3.56–4.90	4.10–5.10	[100]
Bashar I.I., et al.	SFs	0–0.75	30.00–31.35	2.22–2.93	4.33–4.86	[49]
Kumar Y.N., et al.	SFs	0–1.00	43.00–47.05	4.75–6.70	5.75–7.35	[77]
Pham K.V.A., et al.	SFs	0–1.50	32.05–57.02	3.70–4.98	5.89–9.65	[97]
Bellum R.R., et al.	SFs	0–2.50	54.30–61.50	5.62–8.52	7.70–12.15	[103]
Bharath S.R.Y., et al.	PFs	0–1.25	43.20–49.44	4.60–11.80	5.20–14.39	[39]
Wang Y., et al.	PFs	0–2.00	36.57–42.13	2.37–2.85	3.91–5.71	[106]

Key: PPFs is the polypropylene fibers; GFs is the glass fibers; CFs is the carbon fibers; BFs is the basalt fibers; PVAFs is the polyvinyl alcohol fibers; SFs is the steel fibers; PFs is the plant fibers.

**Table 9:** The effect of aspect ratio on geopolymer concrete [97].

Fiber Types	Respect Ratio (L/d)	Fiber Volume Rate (%)	Compressive Strength (MPa)	Splitting Tensile Strength (MPa)	Flexural Strength (MPa)
SFs	60	0–1.50	32.05–57.02	3.70–4.98	5.89–9.65
SFs	70	0–1.50	32.05–59.30	3.70–5.15	5.89–10.22
PPFs	200	0–1.50	32.05–43.33	3.70–4.20	5.89–7.98
PPFs	300	0–1.50	27.13–38.59	3.70–4.11	5.89–7.32

Key: PPFs is the polypropylene fibers; SFs is the steel fibers.

**Table 10:** The effect of hybrid fiber volume rate on geopolymer concrete.

Authors	Fiber Types	Fiber Volume Rate (%)	Compressive Strength (MPa)	Splitting Tensile Strength (MPa)	Flexural Strength (MPa)	Ref.
Aisheh Y.I.A., et al.	PPFs/SFs	0–2.25	115.00–162.00	6.10–8.60	7.60–13.70	[44]
Mousavinejad S.H.G., et al.	PPFs/SFs	0–2.00	97.96–120.98	5.17–7.73	6.79–12.80	[45]
Sukontasukkul P., et al.	PPFs/SFs	0–2.00	35.44–72.99	--	84.90–199.10	[107]

Key: PPFs is the polypropylene fibers; GFs is the glass fibers; SFs is the steel fibers.
